# Psychodynamic Therapies for the Treatment of Substance Addictions: A PRISMA Meta-Analysis

**DOI:** 10.3390/jpm13101469

**Published:** 2023-10-07

**Authors:** Marco Zuccon, Eleonora Topino, Alessandro Musetti, Alessio Gori

**Affiliations:** 1 Department of Health Sciences, University of Florence, Via di San Salvi 12, Pad. 26, 50135 Florence, Italy; marco.zuccon@stud.unifi.it; 2 Department of Human Sciences, LUMSA University of Rome, Via della Traspontina 21, 00193 Rome, Italy; eleonora.topino@gmail.com; 3 Department of Humanities, Social Sciences and Cultural Industries, University of Parma, Borgo Carissimi 10, 43121 Parma, Italy; alessandro.musetti@unipr.it

**Keywords:** substance dependence, substance abuse, psychodynamic therapy, participation in treatment, comorbidities, opiate use disorder, alcohol use disorder, cocaine use disorder, meta-analysis

## Abstract

The aim of this meta-analysis was to compare psychodynamic with other treatments in studies of substance addiction. The literature search was conducted using the PubMed, Web of Science, Cohcrane library, SCOPUS, and Onesearch databases. All studies comparing psychodynamic therapy with other types of psychological interventions for Substance Use Disorder were eligible. Three outcomes were considered to compare intervention performance: substance use, participation in treatment and other symptomatic conditions (OSCs). Hedges’ G was used to measure effect size. The Revised Cochrane Risk of Bias tool for randomized trials was used to assess quality of evidence and possible bias, Egger regression analyses for publication bias, and Q and I-square statistics were used to assess heterogeneity. The alcohol group showed no differences between treatments on the three outcomes. The cocaine group showed no significant differences in the two outcomes, while for OSCs, available data was insufficient. The opioids group showed small but significant differences regarding participation in favor of non-psychodynamic interventions and no significant results for other outcomes. Based on the three measures of recovery considered in this meta-analysis, psychodynamic interventions were shown to be as effective as other psychological treatments in treating substance dependence and proved to be an empirically-supported treatment for the above addictions.

## 1. Introduction

### 1.1. Rationale

Psychodynamic therapy has been used for a broad range of mental health disorders in the last century. From Freud to modern psychoanalysts, ideas have changed, and the range of theories that is currently enclosed has grown disproportionately. Much clinical work has been performed, and many hypotheses have been created on that basis. Despite the abundance of non-experimental literature addressing the efficacy of psychodynamic therapy, assessments that adhere to updated methodological standards are still scarce, and further research is needed [1]. In the field of addiction, the studies of Glower [2], Radó [3], Knight [4] and Simmel [5] were pioneers of a revolutionary psychodynamic vision in this domain. They improve the approach, with new ideas relative to causes, processes and intervening variables within the treatment process. Many others after them have tried to understand the problem of addiction more deeply within the psychodynamic matrix (a good report on the subject, extraneous to the objectives of this work, is available from Yalisove [6] and Potik [7]). However, most of their theories lack adequate and solid methodologically evidence. Only in the last few decades have psychodynamic authors approached evidence-based research and tried to adapt their abstract indicators of efficacy from clinical reports to strict measurements. These modifications made the growth of new supporting evidence possible for evaluations. However, studies relating those works to addiction treatment are few and provide little help in exploring therapy efficacy, especially without having the differences between drugs in mind. Filling this gap and providing a review of those adequate studies is precisely the goal of this meta-analysis.

Looking for evidence of the efficacy of psychodynamic therapies regarding psychological problems as a whole, results are variable between different reviews and meta-analyses [1]. Some authors declare that dynamic treatment is effective for major depressive disorder, panic disorder and borderline personality disorder [8], others observe efficacy for depression, some anxiety disorders, eating disorders and somatic disorders, and in general, it proves to be neither superior nor inferior to the compared interventions [9], and some others have instead found evidence of efficacy for dysthymia, complicated grief, panic disorder and generalized anxiety disorder [10]. One of the most recent meta-analyses on this topic was conducted by Leichsenring and his team [11], who found moderate quality evidence indicating that psychodynamic therapy (PDT) is just as effective as other active therapies for treating depressive, somatic symptoms, anxiety and personality disorders. In 2022, the same author published an umbrella review of recent meta-analyses to explore the efficacy of psychotherapies and pharmacotherapies for mental disorders, finding small effects for both of those protocols [12]. Regarding substance dependence, Gibbons and Crits-Cristoph et al. [8] and Leichsenring et al. [10] report evidence. In the first study, efficacy is highlighted for alcohol and opiate treatment, while in the second study, general efficacy for substance abuse/dependence is reported.

### 1.2. Objectives 

Within the aforementioned psychodynamic framework, the present meta-analysis gathered and compared all studies that refer to substance use treatment and that permit adequate data extraction for efficacy comparisons. This work was organized according to the PRISMA guidelines offered by Liberati et al. [13]. The final outcomes combine data collected with different measurement tools and refer to three areas of the patient’s recovery: quantity of substance use, participation in the proposed activities and severity of the comorbid psychological condition accompanying addiction. The goal is to understand if psychodynamic protocols could be as useful and effective as other treatment protocols already widely covered in the scientific literature. This work focuses specifically on papers that consider and compare psychodynamic therapies with other active psychological treatments from 1970 to 2022, assessing the quality of collected works by the risk of bias, heterogeneity and publication bias, trying to understand how effective those therapies are and trying to know what quality level the evidence reaches in this field. 

## 2. Method

### 2.1. Eligibility Criteria

The Population, Intervention, Comparator, Outcome and Study design (PICOS) framework [13] was used to achieve the objective of evaluating the effects of interventions. Articles were divided based on the main substance of abuse to reduce the effect of different types of treatments. The choice to exclude articles before 1970 from the research process was strictly rooted in the history of psychodynamic therapy itself. Before this date, psychodynamic treatments were not conducted in a way that could permit an objective evaluation of the whole therapeutic process and used techniques not suitable for quantification. This is particularly evident within the addiction field of research [6,7]. 

Population: Patients with a diagnosis of substance use disorders (according to the main diagnostic systems, like DSM and ICD) or subjects in active treatment for it were accepted as eligible for this study. Adults are the focus of this work, so studies were accepted if patient age ranged from 18 to 65 years; 18 and 65 years have been chosen as limits because they are the standard age cutoff between childhood and adulthood, as well as between adulthood and old age, both in the US and Italy. This decision was made to avoid both neurocognitive and physical differences due to growth or ageing and other variables, such as different treatment dynamics, which could be present in child and old patients and not in adult ones that could confound the analysis. For gender, ethnicity and the presence of other diagnoses, no exclusion criteria were added. 

Intervention: All psychodynamic interventions, as described by the authors themselves, and psychotherapies renowned as part of this theoretical category were accepted and gathered. Studies in which psychodynamic therapy was only one of the proposed interventions to the subject were not excluded, but in the assessment of risk of bias, possible risks related to the quality of research due to this type of compound protocol were evaluated, e.g., where patients also participated in other parallel interventions.

Comparator: Comparison groups included in the meta-analysis ranged from renowned psychotherapeutic interventions to more general psychological interventions, like counselling or treatment-as-usual.

Outcomes: To be included, a trial had to use a defined clinical outcome relating to at least one of the three outcomes that were considered, and it must have had enough values to allow for effect size calculations. The choice of outcome fell back to measures of substance use assessments, a report of substance use behavior collected based on a clinical evaluation or physiological exams. Tightly connected with substance use are the experienced levels of other psychological symptoms or comorbidities gathered in the outcome “Other symptomatic conditions”. Last was the “Participation” outcome. Is known that participation in treatment is closely related to therapeutic improvements and that without participation, the chances of improvement are close to zero. Just as the ability to keep patients in therapy represents one of the major problems in their treatment, the effectiveness of keeping them active in it is synonymous with good therapeutic efficacy and must be taken into consideration.

Study design: for the aim of this work, randomized controlled trials, quasi-randomized controlled trials, controlled clinical trials or observational studies where subjects were divided into at least two different types of psychological intervention groups and assessed with comparable quantitative evaluation tools were considered eligible.

### 2.2. Information Sources

The information presented in this paper was gathered from the major scientific databases: PubMed, Web of Science, Cohcrane library, SCOPUS and Onesearch. Brief grey literature research was performed using Google. Some information was collected from books and paper documents. Reference lists were examined from previous meta-analyses to complete the research and include all possible studies consistent with this analysis topic [1,9,10,14,15,16].

All research and gathering processes were conducted from May 2022 to August 2022. For some studies, it was necessary to contact the authors themselves to obtain more information about the measurements that were made. However, these attempts were unsuccessful, leading to the exclusion of these articles from the analyses [17,18,19,20].

### 2.3. Search Strategy

The same search strategy was used for every database consulted. Firstly, preliminary research of titles and abstracts was performed to get an idea of the literature and theories about psychodynamic theory and addiction. Database coverage was limited to 1970 to August 2022:

- Psychodynamic AND (addiction OR substance use disorder)

After this general research, specific terms were added, using the same filters and search conditions with this query:

- (Psychodynamic OR psychoanalytic OR psychodyn* OR psychoan* OR “ego psychology” OR “self-psychology” OR “object relation” OR “intensive expressive” OR “interactional group”) AND (addiction OR substance dependence OR “substance use disorder” OR alcohol OR heroin OR opioid OR cocaine OR marijuana OR hallucinogen OR barbiturates OR methadone OR ketamine OR amphetamines OR methamphetamine OR MDMA) AND (therapy OR psychotherapy OR treatment).

The goal of this strategy was to find every study that took into consideration some psychodynamic-like psychological interventions for patients with some substance use problems. The aim of this phase was not to discriminate between randomized or non-randomized trials or to discriminate between articles with comparisons between therapies and single therapy analysis. Only English articles were considered.

### 2.4. Selection Process

The selection of studies was performed by one reviewer, and these were disclosed in three phases:

- Screening of titles and abstracts;
- Full reading of the articles;
- Analysis of data;

In the first phase, all articles that seemed to evaluate patients with substance use problems that were being treated using psychodynamic treatments were considered. In the second phase, the goal was to understand which articles confronted psychodynamic interventions with non-psychodynamic interventions and collect them for a more specific assessment. In the third phase, the final assessment to evaluate which studies were suitable for this meta-analysis was performed; in other words, it was observed if authors reported valid and useful information that allowed for a statistical analysis of the outcomes. A total of 2198 articles from the various databases were examined, and they were sifted using the software Mendeley (Mendeley Ltd., London, UK), which eliminated duplicates and helped to manage references.

### 2.5. Data Collection Process

As mentioned above, some studies did not have a clear report of patient information or results, and it was necessary to contact the authors themselves to obtain more information about the measurements reported. However, these attempts were unsuccessful, leading to the exclusion of these articles from the analyses [18,19,20,21]. One other problem emerged with multiple reports corresponding to a study. This was the case for Kang et al. [22] and Kang et al. [23], where further answers were not provided.

For those problems, the strategy was to contact authors using the texting tool offered by ResearchGate, whenever authors were registered.

### 2.6. Data Items

From each of the included studies, information was extracted relating to the following: (1) study design, (2) duration of treatment, (3) number of participants, (4) setting, (5) type of substance, (6) diagnostic criteria, (7) age, (8) gender, (9) ethnicity, (10) comorbidities, (11) date of study, (12) number of intervention groups, (13) type of intervention, (14) theory of reference, (15) measured outcomes, (16) time points/follow up, (17) data collection for each intervention group, (18) general conclusions. This information is reiterated in Table S1.

Specifically, eligible outcomes were broadly categorized as follows:

- Substance use:
  Grams/liter per week;
  Reported use or reported abstinence.
- Other symptomatic conditions:
  Pre-post evaluation of co-present psychological problems;
  Assessment of how comorbidities evolve over the time of treatment.
- Participation:
  Participation or non-participation frequency;
  Completer and non-completer report.

In this work, substance use was chosen as the first indicator of treatment efficacy over time. This decision, as well as the other outcome choices, followed past major reviews and meta-analyses. However, according to psychodynamic principles, in most cases, the measurement of use was not representative of a real psychological improvement, and as treatment ended, the patient relapsed, mainly because underlying problems had not been resolved over the time of treatment, just as symptom resolution does not consist of the original trauma. Also, substance use-related behaviors and related problems could be a symptomatic expression of another psychological disorder or could be strictly linked to comorbidities, which are also very frequently encountered in this context [23,24]. This explains the choice to gather and confront information related to other symptomatic conditions not primarily related to the substance use disorder. The last outcome category, but not least in importance, was the participation in treatment. Treatment efficacy and the effective improvement of patients are strictly correlated with the presence of patients during treatment hours. This correlation is well-known among psychologists and explains why this last outcome is so important to collect. An effective treatment of every psychological problem cannot disregard adequate participation that allows for the psychotherapist’s work.

Studies with a minimum follow-up of 3 months or 9 sessions were eligible, even though this seems to be, according to various authors, lower than the minimum time frame required for the effective treatment of this disorder [25,26,27,28], which is in accordance with the average length described by Center for Substance Abuse Treatment [29] for brief substance abuse treatments. No other length or time criteria were used as the efficacy of different approaches was evaluated, either within psychodynamic or other interventions, because every theory has different time rules. Findings related to the participation in treatment indicate that more sessions generally mean more gains, but evidence of inferiority between brief therapies and long therapies has not been found [29].

### 2.7. Study Risk of Bias Assessment

An assessment of the risk of bias in the included studies was performed using the revised Cochrane risk of bias tool for randomized trials (RoB 2.0) [30]. RoB 2.0 addresses five specific domains: (1) bias arising from the randomization process; (2) bias due to deviations from intended interventions; (3) bias due to missing outcome data; (4) bias in the measurement of the outcome; and (5) bias in the selection of the reported result. One author independently applied the tool to each study and recorded supporting information for judgments of the risk of bias for each domain. In case there were any difficulties in assessing the potential for bias or in understanding the possible reasons for these assessments, the author and their supervisor discussed and resolved them through a consensus. Based on Higgins et al. [31], a final evaluation of the risk of bias (low, some concern, high) was conducted. An intention-to-treat assessment of the risk of bias was performed for all three outcomes, and they were collected in three different tables to show the results.

### 2.8. Effect Measures

Acknowledging the great diversity of data types gathered, the guidelines of Hedges et al. [32] were followed. The bias-corrected Standardized Mean Differences (Hedges’ g) and their 95% confidence intervals were selected for the effect size calculation. Hedges et al. [32] also explain the theoretical bases for project analysis and understanding conversions among effect sizes, derived from the different types of data collected from each study. All analyses were performed using the software Comprehensive meta-analysis V3 [30]. The software made it possible to calculate, starting from linear and continuous data, the relative effect sizes based on the Log Odds ratio and Cohen’ D. These were then converted to Cohen’ D to compute the bias-corrected standardized mean difference, i.e., the Hedges’ G. A random-effect model was used for all computations.

If the value of Hedges’ g was negative, the intervention led to lower scores for the patients who received it compared to those in the control group. On the other hand, positive values of Hedges’ g suggest that the intervention was somewhat effective, and the advantage gained from the intervention could be measured. To interpret the size of the effect, the standard threshold was used, rating a small effect = 0.2, a medium effect = 0.4 and a large effect = 0.8 [33].

The large inter-study variability observed while reading the literature prompted us to perform heterogeneity analyses among the studies, so Q and I2 statistics were chosen to evaluate if the observed difference referred to a real difference between studies or due to chance. Large variability explained by real differences would show the need to carry out subgroup analyses among the collected studies.

### 2.9. Synthesis Methods

Given the complexity of the interventions being investigated, included interventions were categorized according to the main type of drug used, forming three groups: alcohol users, cocaine users and opioid users. This categorization led to a reduction in the power of the analysis due to a reduction in the potential articles gathered for statistics, but it was coherent with differences between specific substance treatment protocols, which would represent a big risk of bias for this meta-analysis if considered to be homogeneous.

Only partial differences between control interventions and psychodynamic treatments themselves were assessed only partially, and they were not used for a more in-depth analysis, i.e., those considerations were required in the discussion phase, but they did not lead to any additional analyses. The work aimed to understand if psychodynamic treatment could be useful as treatments already used very frequently for substance-dependent patients and for which there was a big list of research on their effectiveness. Therefore, assuming the general effectiveness of control treatments from the literature, the goal was to observe how psychodynamic treatments perform in comparison to them.

As mentioned earlier, the great diversity of data types made it necessary to calculate the relative effect sizes and then convert them to Cohen’ D and then Hedges’ G for every outcome for each group, as described precisely in chapter 7 of Hedges et al. [32].

All analyses were carried out using the software “Comprehensive meta-analysis v3” [30]. Tables S2–S4 show the data used for statistical analyses with the sample using alcohol, respectively, in relation to the use of substances, participation in treatment and other symptomatic conditions. Tables S5 and S6 instead show data gathered from the cocaine use sample, respectively, in relation to the use of substances and participation in treatment. Other symptomatic conditions with eligible outcomes were reported only in one article. Lastly, in Tables S7–S9 data used for statistical analyses carried out on the opiate user sample were gathered, respectively, in relation to the use of substances, participation in treatment and other symptomatic conditions. For the purposes of the meta-analytic work, as can be seen from the tables, different measurement tools were grouped, based on the category of the result indicator to which the reference was made.

Based on preliminary information gathered from the studies, a random-effects model was chosen for this meta-analysis because the true intervention effects were likely to be diverse across the included studies and therefore not well-captured by a single fixed intervention effect. The random-effects model can better account for the variability in the effect sizes observed across studies, which may be due to factors, such as differences in populations, interventions or outcomes. Additionally, the random-effects model can also help to mitigate the potential impact of small-study effects, which may bias the results of a fixed-effect model. Ultimately, the choice of the meta-analysis model depends also on the acknowledgement of clinical and methodological diversity in the included studies, as well as the research questions being addressed. The manual of Hedges et al. [32] was followed for theoretical guidelines.

Because variability in the effects observed in studies could compromise or influence the interpretations of the obtained results, heterogeneity analysis between studies was conducted. Doing so, it became possible to understand how much of the observed variability was due to chance and how much was due to effective differences between studies. To obtain an estimate of this value, I2 was calculated from the Q statistic.

No additional analysis was planned, like subgroup analysis or meta-regression, even if heterogeneity levels suggested to do so, for reasons of the engagement of the author, who works independently.

No sensitivity analyses were conducted to assess the robustness of the synthesized results, mainly because the majority of articles are at a high risk of bias, and excluding them would lead to an absence of eligible articles.

### 2.10. Reporting Bias Assessment

In addition to the analyses described above, an assessment of the presence of publication bias was also performed. Indeed, it is known that there is a chance that inside the scientific scene, the publication of studies with significant results and large effects became preferable to studies with small or insignificant results. Therefore, studies using a meta-analysis may belong to a niche of lucky studies. The risk associated with this condition is overestimating the true size of the effect, which would be influenced by a biased study sample. To verify the presence of the risk of bias, a visual analysis of the funnel plots was conducted. In these graphs, large sample studies are in the upper part and tend to cluster around the mean effect found. Smaller studies rank at the bottom, due to greater variability in the effect estimates, and tend to be more dispersed across the range of values. In the absence of bias, a symmetrical distribution of studies concerning the size of the effects is expected. In the presence of bias, a set of studies on one side in the lower part of the graph is expected to be seen. This reflects that smaller studies are more likely to be published if they have larger-than-average effects, making them more likely to meet the criterion of statistical significance. To this evaluation, Egger’s regression test was added, which allows for a quantification of the asymmetry and a more precise estimate of the distribution of the studies in the graph.

### 2.11. Certainty Assessment

To evaluate the certainty of evidence for this meta-analysis, it was not possible to use the GRADE approach, due to the variability in outcome measures that had been gathered in the three major outcome categories described. However, information reported in the assessments of certainty could be consulted and was taken into consideration in this meta-analysis, even if not systematically. Study limitations were reported within the risk-of-bias analysis; the consistency of effect recall with the heterogeneity analysis, imprecision, was also taken into consideration and discussed in the “heterogeneity analysis” chapter, indirectness was considered in the “characteristics of studies” chapter, and publication bias was analyzed well in a specific chapter. The certainty of evidence was reported in a narrative form due to the internal limitations, which have just been explained.

## 3. Results

### 3.1. Study Selection

**Figure 1:**
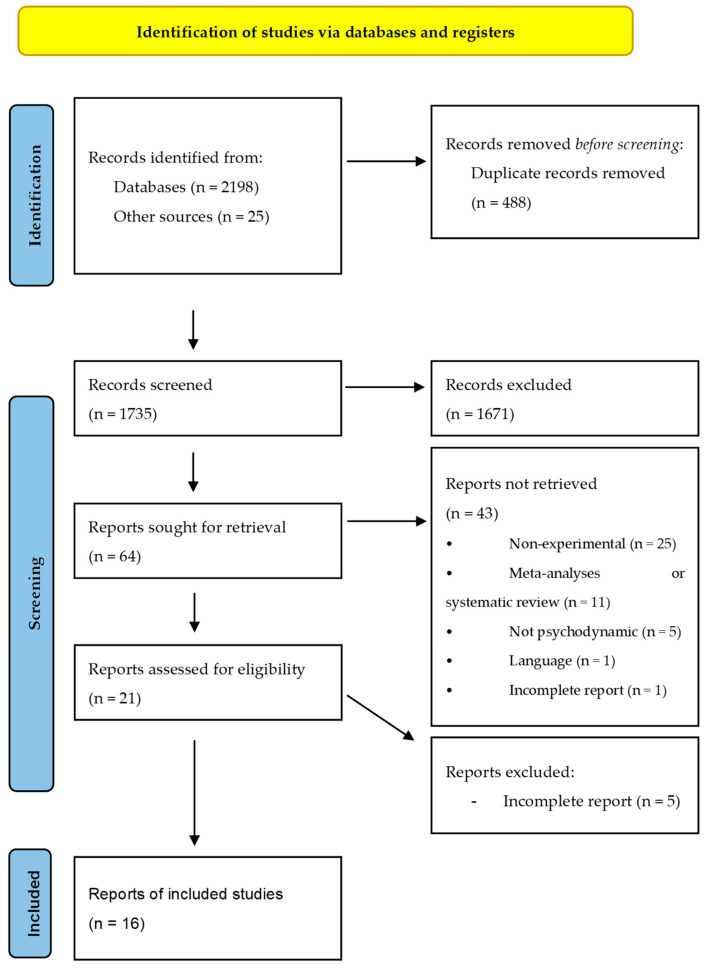
PRISMA flow diagram for the systematic review and meta-analysis, which included searches of databases and other sources.

All selection processes have been summarized in Figure 1. A total of 2223 articles from the various databases were examined; 25 were obtained from paper sources or web searches. After looking for duplicates, 1735 unique articles were evaluated via the abstracts. After this first screening, 64 articles were gathered and read to assess their eligibility. From this second screening process, 43 articles were excluded in this complete reading phase for the following reasons: 25 articles reported descriptions of a theoretical nature or descriptions of clinical cases, 11 articles were meta-analyses or systematic reviews, 5 articles reported non-psychodynamic treatments as experimental conditions, 1 article reported an English abstract but was written in German and 1 article reported incomplete information. In the end, 21 studies were eligible for the systematic review. Five articles were excluded from the analysis because they lacked the necessary information and also after requiring additional input from the authors. A total of 16 studies were ultimately eligible for the meta-analysis and were assessed for their risk of bias (one correction article was excluded from the count and counted as an original article). The characteristics of the 21 studies eligible for the systematic review is found in the chapter on study characteristics. Of the 16 studies taken into consideration for the meta-analysis, one of these [20] was eligible but did not report any distinction regarding the type of substance used, and for this reason, it was excluded from the analysis and was simply briefly described in the final part of the work.

### 3.2. Study Characteristics

In a review examining the performance of psychodynamic therapies in comparison with other substance abuse treatments, the authors included a table presenting, for each included study, the citation, study design, treatment time, sample size, setting, age, sex, substance of abuse, ethnicity, comorbidities, reported outcomes, time points and outcomes assessed for the meta-analysis (Table S1).

### 3.3. Risk of Bias in Studies

A summary of these assessments is provided in Table 1, Table 2 and Table 3.

In terms of the overall risk of bias, there were concerns about the risk of bias for the majority of studies (14/15), with two of these assessed as having a moderate risk of bias and others having a high risk. A text summary is provided below for each of the six individual components of the risk of bias assessment. Risk of bias analysis was conducted and reported for each outcome.

We first analyzed the problem of selective reporting of information by observing which study problems were identified with the reporting of information in general or with the outcomes.

Following the Cochrane risk of bias assessment guidelines, if fewer than 95% of the randomized subjects are present in the results and therefore analyzed for the entire duration of treatment, regardless of whether they have discontinued treatment or not, the study is considered to have a high risk of bias. Many studies in our meta-analysis shared this unfortunate characteristic, as the study population is known for its tendency to drop out of treatment [43] and to suddenly discontinue activities that have already been started (referred to D3 in Table 1, Table 2 and Table 3). This does not justify the lack of data reported in the studies, but it allows us to understand why such a large number of studies fall into this bias. The articles by Ito, Donovan and Hall [35] Rounsaville et al. [36], Sandahl et al. [37], Woody et al. [19], Ojehagen et al. [38] and Hoyer et al. [42] fall into this problem. In these cases, it was not possible to exclude that the results were influenced by these omissions and, in particular, with respect to the most severe conditions.

Domain 2 reports variable results, with a similar rate of high, some concern and low risk. This refers to the “definition of the intended intervention” and a lack of high evidence because the majority of experimental designs combined psychological with medical, pharmaceutic or other psychological treatments that could not always be under strict control during the time of the study; however, this is required to reach the good methodological standard.

Regarding the reporting of outcome measures, as evaluated in D4 and D5, the studies obtained positive results, with few assessments of “some concern” and a large amount of low risk of bias. In general, it was found that the measures reported in the results section were consistent with those declared in the methodological and protocol section and those were coherent with the constructs that have been measured. Except in one case [14], in which the research objectives were specified in a preliminary article, and with which it was possible to check the actual presence of all the declared measures, for the other studies, this assessment had to be based on the information contained in the articles themselves.

The randomization process, as explored by domain 1, collected generally good results, but in some cases, this process was not well described and the evaluation fell into the inferior quality category as “some concern” or “low quality”, affecting the final evaluation.

### 3.4. Results of Individual Studies

**Figure 2:**
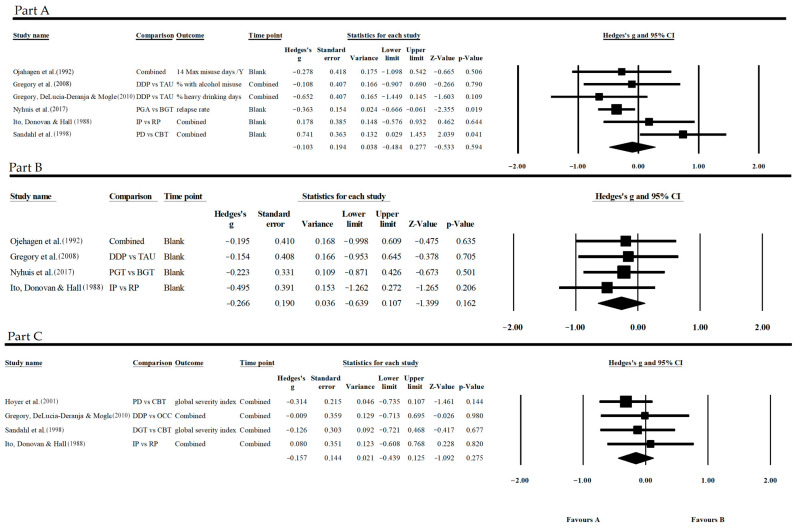
Forest plot of “Alcohol—Substance Use” (**Part A**), “Alcohol—Participation” (**Part B**), and “Alcohol—Other Symptomatic Conditions” (**Part C**). References: Ojehagen et al. [38], Gregory et al. [34], Gregory, DeLucia-Deranja & Mogle [27], Nyhuis [36], Ito, Donovan & Hall [35], Sandahl et al. [37], Hoyer et al. [42].

Looking for “substance use” in the alcohol group (Figure 2, Part A), data from six studies were gathered. Specifically, the effect variability between the studies was quite wide, even if only in two cases was this significant, and this showed interesting differences in favor of CBT or dynamic treatment. The studies of Ito, Donovan and Hall [35] and Sandahl et al. [37] shared the same control treatment, i.e., a treatment of a cognitive behavioral nature based on the model devised by Marlatt [44,45], called the Relapse Prevention model. Ojahagen [38] also used a therapy within the CBT paradigm, but it was based on another model, namely the multimodal theory, described by Lazarus [46], and in Nyhuis et al. [36], another CBT-based treatment was found, the Combined Behavioral Intervention, as described by Longabaugh et al. [47]. Instead, regarding treatment-as-usual, as described in Gregory et al. [27,34], there was not enough information to trace it back to a specific theory. In the literature, this type of intervention is generally described as a set of cognitive behavioral techniques and the 12-step program; however, a more in-depth analysis [48] showed how the theoretical categorization of these procedures was much more complex and multi-component in clinical practice.

Based on the results obtained by Sandahl et al. [37], it appears that the Relapse Prevention model of treatment reduces alcohol consumption more than dynamic treatment, and the difference is significant (G = 0.741, *p* = 0.041, CI = 95%). However, these data are not supported by other studies. Looking for other types of treatment, whether they originate from the CBT paradigm or fall within the description of treatment-as-usual, no significant differences have been reported. In contrast with these results, Nyhuis et al. [36] reported a slight but significant difference that promotes the dynamic treatment for the reduction of intake (Figure 2, Part A); however, the effect size was relatively small, indicating only modest differences (G= −0.363, *p* = 0.019, CI = 95%).

Both with regard to participation (Figure 2, Part B) and other symptomatic conditions (Figure 2, Part C), the individual studies did not report significant differences between interventions.

**Figure 3:**
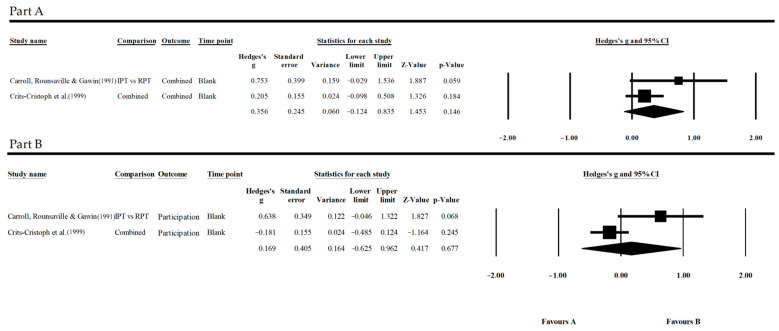
Forest plot of “Cocaine—Substance use” (**Part A**) and “Cocaine—Participation” (**Part B**). References: Carrol, Rounsaville & Gawin [39], Crits-Cristoph et al. [14].

Concerning the use of cocaine (Figure 3, Part A), it was possible to collect data on the trend in its use in patients under treatment for only two studies. Although two studies are sufficient to conduct a meta-analysis, this limits generalizations. The main difficulty that limited the number of eligible articles was the quality of the outcome report, often without traceable data for the meta-analysis.

Crits-Cristoph et al. [14] compared the dynamic treatment to three different types of therapy, so the effect size is the average of the differences observed between these and the dynamic therapy used. Both in the papers of Crits-Cristoph et al. [14] and Carrol, Rounsaville and Gawin [39] no significant trends were found. In the first study, differences were not significant, except for “individual counseling”, which showed better, statistically significant results compared to “supportive expressive” dynamic therapy. In the second study, results were also not significant, and no evidence was found in favor of either (G = 0.753, *p* = 0.059, CI = 95%).

Concerning participation in the treatment (Figure 3, Part B), the differences in the comparisons made by Crits-Cristoph et al. [14] and Carrol, Rounsaville and Gawin [39] proved to be not significant. These results showed an effective equivalence between observed treatments. One interesting result that emerged from Crits-Cristoph et al. [14] is the significant superiority of both dynamic and CBT interventions to individual and group counselling, in contrast with the result within the “substance use” outcome.

Concerning the “symptomatic condition” outcome, only Crits-Cristoph et al. [14] reported data that could be used, while Carrol, Rounsaville and Gawin [39] and the other articles did not consider this outcome or did not report the achieved results. From the work of Crits-Cristoph et al. [14], it is possible to conclude that, besides the treatment of choice, the observed rate of psychological symptoms tends to decrease together with a decrease in substance use. These results are in line with those reported by NIMH on the co-occurrence of psychiatric disorders and substance addiction, showing how these often support each other [49], and would confirm the results of analyses previously carried out on this topic with reference to other symptomatic conditions, such as depression [50].

**Figure 4:**
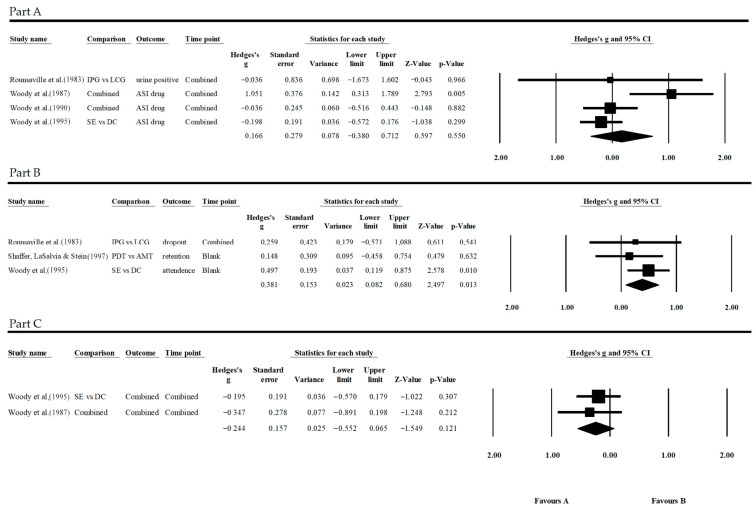
(**Part A**) Forest plot of “Opiates—Substance Use”, (**Part B**) “Opiates—Participation”, and (**Part C**) “Opiates—Other Symptomatic Conditions”. Referenes: Rounsaville et al. [40], Woody et al. [52], Woody et al. [51], Woody et al. [19], Shaffer, LaSalvia & Stein [17].

Concerning opioid use (Figure 4, Part A), data were available from four studies, three of which were conducted by the same authors, one as a follow-up study and the other as a validation study. The three studies by Woody et al. show conflicting results; Woody et al. [41] are reported here as Woody et al. [51] as some data reported erroneously in 1983 have been highlighted and corrected later by the authors, so Woody et al. (1987) [52] is reported as a follow-up study of corrected Woody et al. (1990) [51]. Non-dynamic treatments used as comparisons, drug counseling and cognitive treatment, had no reported significant differences from the expressive supportive treatment. In the follow-up study [52], on the other hand, two and a half years after the start of treatment, the non-dynamic treatments resulted in a significantly higher reduction in consumption than the supportive-expressive treatment. This difference was very large, in addition to being statistically significant (G = 1.051, *p* = 0.005, CI = 95%). In the validation study by Woody et al. [19], only drug counselling was considered as the control condition. In this case, as in the first study, the differences between treatments were minimal and not significant. In Rounsaville et al. [36] instead, both interventions led to an increase in the use of substances over time, so it is possible to state that the two treatments proved to be equally ineffective in reducing opiate consumption.

Concerning treatment participation (Figure 4, Part B), data from three studies were collected. In all three cases, whether they were alternative methadone treatment by Shaffer, LaSalvia and Stein [18], drug counselling by Woody et al. [19] or the low-contact treatment used in Rounsaville et al. [40], no significant differences were recorded. The only exception was Woody et al. [19], where a medium-sized statistically significant difference in favor of the control condition was reported (G = 0.497, *p* = 0.0010, CI = 95%).

Regarding the symptomatic improvement associated with the treatment of opioid use (Figure 4, Part C) differences favorable to one treatment could not be observed in both studies, i.e., Woody’s follow-up study [52], which also reported the original data from Woody et al. [41], and the 1995 validation study [19] from the same authors. There they found no significant differences among dynamic treatment, drug counselling and cognitive treatment.

### 3.5. Results of Syntheses

#### 3.5.1. Risk of Bias

Looking for common problems in the assessment of the risk of bias, the inclination to suddenly interrupt the activities started, and specifically, the treatments heavily influenced the evaluation of articles. This affected the evaluation of the risk of bias which, according to the Cochrane protocols, is at a high risk if this percentage of abandonment exceeds 5%. Much higher percentages characterize many studies: Ito, Donovan and Hall [35], Rounsaville et al. [40], Sandahl et al. [37], Woody et al. [19], Ojehagen et al. [38] and Hoyer et al. [42]. Other problems that refer partially to the interruption of treatments are deviations from the intended intervention, where patients could not provide a clear report of their treatments over time and compromise the evaluation of chosen treatments. Again, within the domain “missing outcome data”, the same studies provide low-quality information, due to the low completers ratio or incomplete reporting of it. Generally, as previously reported in tables, most studies concerning this topic are at a high risk of bias, and only three studies could be considered as having a low risk of bias or some concern. This last evaluation refers to an incomplete description of the randomization process.

#### 3.5.2. Alcohol Results

As was previously seen for the individual studies, the results were very variable in the various conditions taken into consideration. In this section of the work, results of meta-analyses and statistics will be reported and analyzed.

Within the alcohol sample, all three outcomes considered showed no significant. The slight tendency that emerged was not significant, and these differences could be due to chance, both when we evaluate the effectiveness in reducing substance use, for the ability to keep subjects in treatment, and for the effectiveness in minimizing the pathological symptoms that accompany addiction:

- Substance use: G = −0.103, *p* = 0.594, CI = 95%;
- Participation: G = −0.266, *p* = 0.162, CI = 95%;
- Other symptomatic conditions: G = −0.157, *p* = 0.275, CI = 95%.

#### 3.5.3. Cocaine Results

As for the cocaine group of studies, only two possible assessments were possible, as the *Other symptomatic condition* was only clearly stated in one study. From the scant evidence that emerged about the treatment of cocaine use, it was not possible to highlight a favorable trend for dynamic or non-dynamic treatments for both outcome use and outcome participation. In both cases, these differences were not statistically significant, although for *Substance use*, this condition was very close:

- Substance use: G = 0.276, *p* = 0.055, CI = 95%;
- Participation: G = 0.169, *p* = 0.677, CI = 95%.

#### 3.5.4. Opioid Results

Within the opioids group of studies, only significant results of the meta-analysis related to *Participation* emerged. For *Substance use*, the observed effect was practically equivalent among treatments. The measurement of participation proved to be significant for the non-dynamic treatments, although this effect was relatively small to be seen as a possible favorable trend. The *Other symptomatic condition* did not show a significant difference:

- Substance use: G = 0.025, *p* = 0.855, CI = 95%;
- Participation: G = 0.381, *p* = 0.013, CI = 95%;
- Other symptomatic condition: G = −0.244, *p* = 0.121, CI = 95%.

Summarizing the conclusions highlighted by the study, we can deduce how, in general, the results show how the dynamic treatment performed in a similar way, or in any case not being statistically significant, compared to other types of treatment used in the assessment of consumption, participation and symptomatic conditions. These results, however, must also be interpreted in light of the differences between the studies, and to do this, analyses have been carried out to assess the heterogeneity of the studies under consideration, which we will see in a later chapter.

### 3.6. Heterogeneity

For alcohol outcomes, heterogeneity was not found among effect sizes either in the Q and *p*-value analysis or the I2 analysis (Table 4).

Describing the assessment of the heterogeneity in the “cocaine” condition (Table 5), a low–moderate heterogeneity in relation to the “use” outcome and a high one about the “participation” outcome was shown, since the two studies taken into consideration showed the opposite results. An assessment of “Other symptomatic conditions” was not possible due to the failure to achieve the minimum number of studies required.

Concerning the analysis of the “opiates” condition (Table 6), a moderately high degree of heterogeneity regarding the “use” outcome was shown, whereby the observed differences would appear to be due to real differences between the studies, which would therefore make them hardly comparable.

### 3.7. Publication Bias

The small number of studies collected makes this type of assessment less reliable compared to larger meta-analyses; however, it could be useful to obtain a better understanding of the evidence state. For “use” and “participation” outcomes related to alcohol use, the observed asymmetry was relatively low, both graphically and from Egger’s test, while it reported an intercept value of 2.43 and *p* = 0.01 for the “other symptomatic conditions” outcome, which indicates a possible publication bias. Indeed, smaller studies with large effect sizes may have been overrepresented in the performed analyses. Referring to cocaine condition outcomes, unfortunately, it was not possible to carry out the publication bias analysis because the minimum number of studies, i.e., 3, was not gathered. An analysis of the risk of publication bias for the condition “opioids” was performed, where permitted, based on the number of studies, for the outcome “use” and “participation”. In both cases considered, no publication bias was observed either with a visual analysis or with Egger’s test.

### 3.8. Certainty Assessment

Study limitations are numerous, as described in the risk of bias analysis; in general, for all three outcomes, a clear high-risk report was visible. Unfortunately, this was due to specific limitations and problems within the psychological meta-analytic framework and within the sample population analyzed. Regarding the consistency of effect recall with the heterogeneity analysis, in this work, inconsistency emerged from Cocaine-Participation, where this was due to differences in treatment protocols between studies, and it emerged from Opiates-substance use, where protocols that have been compared are also largely different. Imprecision was also taken into consideration and, starting from the effect size measurements obtained, it was difficult to define the results collected here as “precise”, largely due to insignificant results. Indirectness was taken into account indirectly in the “characteristics of studies” chapter and achieved a high-risk rating due to the previously mentioned large differences between the collected studies. Lastly, the publication bias was comprehensively analyzed in the dedicated chapter; however, no major problems related to this domain were identified.

## 4. Discussion

The purpose of this meta-analysis was to examine how psychodynamic treatments perform compared to other psychological treatments. The aim of these comparisons is to explore whether the evidence in the literature allows for one type of treatment to be favored over the other and therefore indicate a possible more advantageous path to follow in the treatment of this patient population.

In general, no significant differences were found between treatments. As far as alcohol is concerned, for all three outcomes taken into consideration, the observed differences from the data analysis do not appear to be significant in any case. As far as cocaine is concerned, only two evaluations were possible, as the symptomatic condition outcome was exposed only in one study. From the little evidence that emerged about the treatment of cocaine use, results highlight the insignificant differences in treatments both for the consumption outcome and for the participation outcome. As far as opiates are concerned, on the other hand, the most heterogeneous results between the conditions can be seen. Looking at “Substance use”, no differences between treatments were observed. In the “Other symptomatic condition”, differences were not significant, while for the measure of participation, the non-dynamic treatments proved to be statistically superior to dynamic treatment, although this effect was relatively small.

These results could confirm the various observations according to which it was not the techniques used that make the difference, but what really matters is that the people involved in the therapeutic relationship believe in what they are implementing and create a good therapeutic alliance, as stated by various researchers, which follows the “common factor” or “contextual” model of psychotherapy [53,54].

In most comparisons, the dynamic treatment proved to be as effective as other treatments, i.e., other psychological therapies or specific interventions for substance-dependent patients. Furthermore, a slightly greater effect for the Relapse Prevention protocol compared to dynamic therapy within alcohol use treatment has been observed [37]; however, this is not corroborated by other studies, like Ito, Donovan and Hall [35], where the study’s findings showed that the Relapse Prevention protocol is just as effective as other treatment approaches. Other paradigms did not show convincing evidence to tip the assessment to one side or the other in this meta-analytic work. However, psychotherapy works and, looking at specific results in gathered studies, psychodynamic-based treatments appear to be equal [19,52] or slightly superior [14] to non-psychotherapy treatments, like counselling; equal to Cognitive-Behavioral Treatment [14,19,37,42]; and report no differences with the treatment-as-usual intervention [27,34]. As described by Santa Ana et al. [48], this includes a mix of different strategies from different psychotherapy and psychosocial theories and focuses on the reference problem, so it is based on principles shared by those of psychotherapies.

These results indicate, once again, that a general equivalence in the efficacy of the treatments that engage psychotherapeutic principles emerges from the analyses. These are consistent with other precedent reviews and meta-analyses on psychodynamic therapy efficacy [7,9]. Two other important aspects that emerge from the review of different studies are that the majority of experimental designs combined psychological with medical and pharmaceutic treatment. According to Carrol [55], who expresses the necessity of integrated psychological and pharmacological treatments, the combination of these two interventions is necessary to improve the chance of a successful recovery. This result was also confirmed in other two studies [56,57]; however, the literature on this topic is very broad and tends to agree. The last aspect pointed out is the very high rate of multiple diagnoses in this population, which complicates interventions and adds another layer to overcome toward recovery. This is visible in studies gathered for this meta-analysis and is coherent with previous findings on the high rate of multiple diagnoses in a substance-dependent population [58,59,60].

To summarize the conclusions highlighted by the study, psychodynamic treatment performance does not differ significantly from other types of treatment, like cognitive-behavioral interventions or drug-specific counseling in regard to drug consumption, participation in treatment and the severity of other symptomatic conditions. Only one significant substantiation was found; however, the effect is limited and little. However, more complex themes emerge besides the statistical meta-analytic work, highlighting the dropout problem, comorbidity mental illness and integration of treatment, which need to be acknowledged to improve therapies and develop innovative solutions to problems that have become part of the experience of everyday addiction centers.

### Limits

This work presents limitations that should not be underestimated. First, the quality of the evidence is not extremely high. Unfortunately, dropouts are frequent in this population [34], and it is difficult to have patients who are not attending multiple therapies, as investigated articles showed, so clear results free from possible bias are difficult to reach using common tools shared by the scientific community, such as the Cochrane tool for a risk of bias assessment. This could affect the risk of bias evaluation and consequently the value of the final conclusions. However, these problems make the difficulties encountered by researchers on this topic evident. For the majority of gathered studies, methodological and data reporting problems have been found. The lack of specificity in the protocol descriptions and multiple uncontrolled treatments combined with a high dropout rate make it unlikely that the results will achieve the quality necessary to be a credible representation of substance-dependence treatments. However, since abandonment in the therapy of these patients has always been reported and most of the theoretical treatises on therapy for substance addicts mention this type of problem, we can consider these results as relating to the small percentage of patients who, once a therapy has been started, can complete it. That is not far away from the normality of substance-dependence treatment services according to a recent meta-analysis [34] and affects one of the fundamental domains for the evaluation of the quality of the evidence.

As previously mentioned, the effectiveness of therapy strongly depends on the amount of time and interest dedicated to it, so starting from these bases, we can interpret the results as the possible outcomes that a subject could encounter once they have started and completed the treatment, which therefore is not synonymous with certain improvements but which can certainly give concrete possibilities if performed correctly by the therapist and the patient. For those who abandon, the probability of improvement is very low, and if there is this, it is difficult to conclude on the extent of the underlying problem and its true nature. In addition to this limitation, the large differences observed between the studies do not allow for specific comparisons to be made between different therapies, and it has not been possible to carry out further analyses capable of detecting the superiority of one specific type of treatment over another, so the analysis remained at a superficial level of a description of “dynamic versus non-dynamic”. These differences are also reflected in the type of outcome recorded in the various studies, and for this reason, the categories of outcomes were also chosen arbitrarily, and the comparisons reflect the author’s hypotheses and the possibilities given by statistics, rather than the effective comparability of the outcome measurement indices in terms of the specificity of the variable measured. Furthermore, the fact that the entire meta-analysis process was conducted by a single author, without the possibility of comparing possible inconsistencies in the evaluation, increases the risk that the work presents defects in terms of accuracy. The last limit, but no less important, is the exclusion of articles in languages other than English and the only superficial search of the so-called grey literature, i.e., those articles, which, for various reasons, have not been published and which could instead have reported different conclusions to those observed, as discussed in the section on publication bias.

## 5. Conclusions

Through a synthesis of the existing scientific literature, this work supported the need for a more accurate work which, as performed by NIDA for Crits-Cristoph et al. [14], allows for a conclusion that is free from the risk of bias and possible interfering factors. The only significant results, which lead the conclusions to lean in favor of a type of treatment, are those relating to participation in the treatment of cocaine use, which, however, are not corroborated by observable improvements in the other two indices considered. To date, it seems that the evolution of dynamic treatments has made it possible to maintain a comparable level of improvement in the patient’s problems compared to other therapies, thus indicating the possibility of using these treatments as alternatives to non-dynamic ones. In conclusion, these data seem to indicate that psychodynamic treatment represents an evidence-based psychotherapy for substance addictions.

The richness of these results lies in their practical implications, since a better comprehension of which therapy works better for a specific type of patient permits us to guarantee better care and reduce dropouts and relapses with reduced frustration for patients, therapists and services. Achieving these objectives is therefore very important at all levels of treatment and should not be underestimated. Greater evidence in favor of specific therapies also allows for further steps forward in understanding how psychotherapy works, favoring the possible integration based on the functionality of the interventions constructed subjectively based on the individual patient with his specific features.

## Figures and Tables

**Table 1:** Risk of bias analysis for articles with the “Substance use” outcome.

Reference	Substance	D1	D2	D3	D4	D5	Overall
Gregory et al. [34]	alcohol	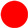	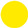	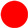	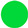	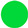	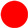
Gregory, DeLucia-Deranja and Mogle [27]	alcohol	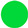	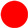	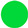	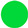	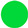	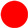
Ito, Donovan and Hall [35]	alcohol	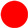	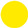	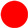	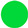	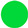	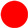
Nyhuis et al. [36]	alcohol	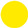	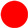	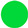	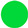	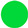	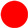
Sandahl et al. [37]	alcohol	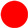	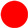	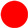	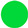	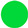	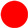
Ojehagen et al. [38]	alcohol	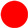	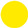	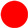	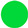	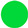	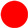
Carroll, Rounsaville and Gawin [39]	cocaine	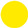	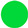	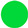	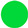	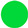	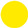
Crits-Cristoph et al. [14]	cocaine	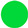	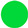	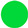	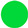	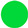	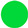
Rounsaville et al. [40]	opiates	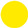	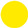	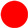	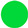	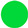	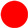
Shaffer, LaSalvia and Stein [17]	opiates	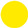	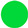	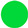	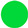	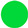	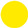
Woody et al. [41]	opiates	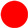	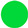	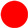	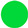	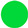	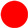
Woody et al. [19]	Opiates	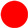	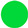	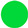	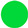	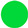	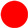

Note: D1 randomization process, D2 deviations from the intended interventions, D3 missing outcome data, D4 measurement of the outcome, D5 selection of the reported result. GREEN low risk; YELLOW some concerns; RED high risk.

**Table 2:** Risk of bias analysis for articles with the “Participation” outcome.

Reference	Substance	D1	D2	D3	D4	D5	Overall
Gregory et al. [34]	alcohol	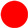	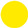	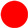	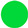	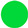	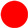
Ito, Donovan and Hall [35]	alcohol	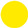	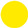	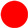	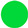	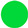	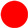
Ojehagen et al. [38]	alcohol	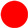	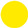	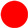	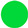	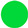	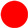
Gregory, DeLucia-Deranja and Mogle [27]	alcohol	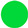	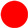	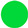	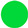	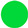	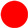
Carroll, Rounsaville and Gawin [39]	cocaine	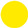	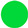	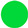	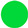	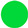	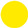
Crits-Cristoph et al. [14]	cocaine	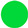	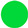	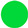	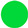	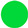	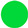
Rounsaville et al. [40]	opiates	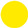	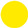	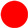	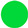	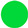	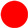
Shaffer, LaSalvia and Stein [17]	opiates	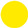	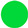	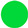	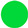	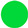	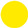
Woody et al. [19]	opiates	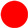	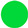	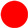	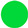	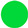	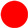

Note: D1 randomization process, D2 deviations from the intended interventions, D3 missing outcome data, D4 measurement of the outcome, D5 selection of the reported result. GREEN low risk; YELLOW some concerns; RED high risk.

**Table 3:** Risk of bias analysis for articles with the “Other symptomatic conditions” outcome.

Reference	Substance	D1	D2	D3	D4	D5	Overall
Gregory et al. [34]	alcohol	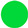	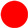	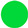	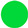	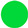	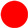
Gregory, DeLucia-Deranja and Mogle [27]	alcohol	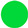	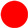	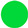	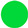	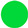	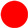
Hoyer et al. [42]	alcohol	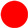	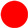	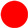	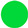	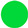	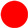
Ito, Donovan and Hall [35]	alcohol	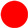	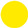	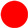	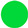	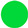	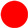
Sandahl et al. [37]	alcohol	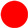	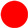	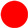	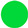	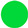	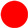
Gregory et al. [27]	alcohol	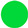	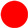	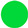	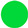	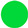	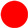
Carroll, Rounsaville and Gawin [39]	cocaine	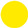	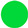	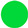	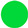	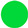	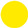
Crits-Christoph et al. [14]	cocaine	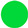	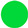	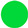	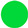	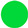	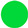
Rounsaville et al. [36]	opiates	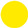	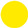	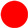	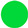	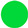	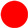
Woody et al. [41]	opiates	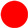	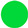	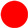	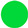	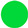	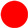
Woody et al. [19]	opiates	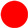	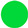	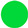	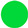	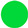	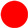

Note: D1 randomization process, D2 deviations from the intended interventions, D3 missing outcome data, D4 measurement of the outcome, D5 selection of the reported result. GREEN low risk; YELLOW some concerns; RED high risk.

**Table 4:** Heterogeneity analyses of outcomes. Q and I-squared statistics were used.

Outcome	Q-Value	Df (Q)	*p*-Value	I-Squared
Substance use	10.10	5.00	0.07	50.52
Participation	0.46	3.00	0.92	0.00
Other symptomatic conditions	1.16	3.00	0.76	0.00

**Table 5:** Heterogeneity analyses of outcomes. Q and I-squared statistics were used.

Outcome	Q-value	Df (Q)	*p*-Value	I-Squared
Substance use	1.667	1.00	0.196	40.015
Participation	4.581	1.00	0.032	78.172

**Table 6:** Heterogeneity analyses of outcomes. Q and I-squared statistics were used.

Outcome	Q-Value	Df (Q)	*p*-Value	I-Squared
Substance use	8.867	3.00	0.031	66.168
Participation	1.014	2.00	0.602	0.00
Other symptomatic conditions	0.206	1.00	0.649	0.00

## Data Availability

Not applicable.

## Supplementary Materials

The following supporting information can be downloaded at: https://www.mdpi.com/article/10.3390/jpm13101469/s1, Table S1: Extracted information for each reported study. Study design, treatment, time of the intervention, number of participants, setting, substance of abuse, age, sex, ethnicity, date of the study, type of intervention, reported outcome, follow-up and outcome available for the aim of this meta-analysis have been reported. Table S2: Statistical analyses carried out on the sample using alcohol, respectively, in relation to “substance use”. Table S3: Statistical analyses carried out on the sample using alcohol, respectively, in relation to “participation”. Table S4: Statistical analyses carried out on the sample using alcohol, respectively, in relation to “other symptomatic conditions”. Table S5: Statistical analyses carried out on the sample using cocaine, respectively, in relation to “substance use”. Table S6: Statistical analyses carried out on the sample using cocaine, respectively, in relation to “participation”. Table S7: Statistical analyses carried out on the sample using opiates, respectively, in relation to “substance use”. Table S8: Statistical analyses carried out on the sample using opiates, respectively, in relation to “participation”. Table S9: Statistical analyses carried out on the sample using opiates, respectively, in relation to “other symptomatic conditions”.
