# Supplementary material for: Psychodynamic Therapies for the Treatment of Substance Addictions: A PRISMA Meta-Analysis
Source: J Pers Med. 2023 Oct 7;13(10):1469. doi: 10.3390/jpm13101469 (PMC10608724; doi:10.3390/jpm13101469)
Supplement: Supplementary file 1 [file jpm-13-01469-s001.zip › jpm-2614929-supplementary.pdf]

SUPPLEMENTARY TABLES

Table S1: extracted information for each reported study. Study design, treatment, time of the intervention, number of participants, setting, substance of abuse, age, sex, ethnicity, date of the study, type of intervention, reported outcome, follow up, outcome available for the aim of this meta-analysis have been reported.

**SOURCES**	STUDY DESIGN	TREATMENT TIME	SUBJECTS	SETTING	SUBSTANCE	AGE	SEX	ETHNICITY	COMORBIDITIES	TREATMENTS	REPORTED OUTCOMES	TIME POINTS	OUTCOMES (• MEAN PRESENCE)
Carroll, Rounsaville & Gawin [40]	Randomized Controlled Trial	3 months	42	outpatient	cocaine	18-35 IPT: 26.8 (8.8) RP: 26.6 (4.0)	IPT: 17 M 4 F RP: 14 M 7 F	whites: 18 IPT 14 RP blacks: 2 IPT 7 RP Hispanic: 1 IPT 0 RP	Depressive Disorders: 4 IPT 4 RP Generalized Anxiety Disorder: 0 IPT 1 RP antisocial personality disorder: 5 ITP 7 RP alcoholism: 7 IPT 6 RP	• relapse prevention (21) • interpersonal psychodynamic therapy (21)	• abstinence and cure rates (measured in "short-term abstinence": at least 3 weeks of abstinence over the 12 weeks; and "short-term cure": at least 3 weeks of abstinence at the end of treatment) • severity of drug use (Addiction Severity Index, Cocaine Craving and Use Scale, urine test) • severity of psychiatric symptoms (Schedule for Affective Disorders and Schizophrenia for Lifetime Occurrence)	CCUS weekly, ASI every month. In general, the other indices were measured before treatment and at the end of 12 weeks.	SUBSTANCE USE: • PARTECIPATION: • SYMPTOMATIC CONDITIONS: X

Crits-Christoph et al [15]	Randomized Controlled Trial	12 months	487	outpatient	cocaine	18-60 SE: 38.8 CT: 40.0 IDC:40.1 GDC: 42.6	SE: 9 M 4 F CT: 12 M 3 F IDC: 4 M 8 F GDC: 8 M 2 F	White: 12 SE 14 CT 9 ICT 8 GDC Afro-American: 0 SE 1 CT 3 ICT 1 GDC other: 1 SE 0 CT 0 ICT 1 GDC	33% alcohol addiction 4.5% cannabis addiction 17% abuse of cannabis 40% antisocial personality disorder 28% cocaine-induced mood disorder 4.9% cocaine-induced anxiety disorder	• cognitive psychotherapy + group counseling for addictions (119) • supportive-expressive therapy + group counseling for addictions (124) • individual counseling for addictions + group counseling for addictions (121) • group counseling for addictions (123)	• substance use (Addiction severity index, self-report on cocaine use, weekly urine tests) • psychopathology (California Psychological Inventory Socialization Scale, Hamilton Rating Scale for Depression, Beck Anxiety Inventory, Brief Symptom Inventory, ASI-Psychiatric Severity Composite score) • therapeutic alliance (Helping Alliance Questionnaire, California Psychotherapy Alliance Scale)	measurements at baseline, 3, 6, 9 and 12 months for all assessments. Up to 6 months for BAI, HRSD and IIP	SUBSTANCE USE: • PARTECIPATION: • SYMPTOMATIC CONDITIONS: •
Gregory et al [36]	Randomized Controlled Trial	12 months	30	outpatient	alcohol	18-45 DDP: 28.3 (7.1) TAU: 29 (8.6)	DDP: 2 M 13 F TAU: 4 M 11 F	Native Americans: 1 DDP 0 TAU blacks: 1 DDP 0 TAU Hispanic or Latin: 0 DDP 1	43% antisocial personality disorder 17% bipolar disorder 67% alcohol addiction 33%	• dynamic deconstructive psychotherapy (15) • treatment as usual (15)	• parasuicidal behavior (Lifetime Parasuicide Count) • improper use of alcohol (Addiction Severity Index) • institutional assistance (Treatment History Interview) • depression (Back	pre-study measurements and at 3, 6, 9, 12 months	SUBSTANCE USE: • PARTECIPATION: • SYMPTOMATIC CONDITIONS: •

								TAU Caucasian: 13 DDP 14 TAU	alcohol abuse		Depression Inventory) • dissociation (Dissociative Experiences Scale) • social support (Social Provision Scale) • BPD severity (Borderline Evaluation of Severity over Time)		
Gregory et al [28]	naturalistic follow up	12 months	16	outpatient	alcohol	18-45 Same as Gregory (2008)	Same as Gregory (2008)	Same as Gregory (2008)	Same as Gregory (2008)	• dynamic deconstructive psychotherapy (8) • treatment as usual (8)	• parasuicidal behavior (Lifetime Parasuicide Count) • improper use of alcohol (Addiction Severity Index) • institutional assistance (Treatment History Interview) • depression (Back Depression Inventory) • dissociation (Dissociative Experiences Scale) • social support (Social Provision Scale) • BPD severity (Borderline Evaluation of Severity over Time)	30 months	SUBSTANCE USE: • PARTECIPATION: • SYMPTOMATIC CONDITIONS: •

Hoyer et al [40]	natural observational study	14 weeks	107	inpatient	alcohol	42.5 mean	50% F 50% M	NA	NA	• psychodynamic therapy (45) • cognitive-behavioral therapy (49)	• conflict evaluation (Lauterbach: The measurement of personal conflict; Lauterbach, Newman: Computerizing intrapersonal conflict formulation method [62]) • severity of symptoms (revised Symptom check list, Global Severity Index) • psychological well-being (Mood Survey, Satisfaction with Life Scale)	first or second, fifth or sixth, ninth or tenth, thirteenth or fourteenth week of treatment	SUBSTANCE USE: X<br>PARTECIPATION: X<br>SYMPTOMATIC CONDITIONS: •
Ito, Donovan & Hall [36]	Controlled Clinical Trials	6 months	49	inpatient aftercare	alcohol	RP:35 (8.5) IP: 38 (11.25)	100% M	NA	NA	• cognitive-behavioral group for relapse prevention (20) • interpersonal group psychotherapy (19)	• alcohol use (Health and Daily Living Form, Alcohol Dependence Scale, structured interviews and collateral reports) • process of psychological change (Self Efficacy scale - temptation and self-efficacy, Coping Behaviors	pre-treatment measurements, at the end of treatment (8 weeks) and 6 months follow-up	SUBSTANCE USE: •<br>PARTECIPATION: •<br>SYMPTOMATIC CONDITIONS: •

											Inventory, structured interviews and collateral reports) • participation in the aftercare program		
Nyhuis et al [37]	Quasi-Randomized Controlled Trial	6 months	215	outpatient	alcohol	49.6 (10) PIT: 50.5 (10) CBI: 48	PIT: 62 M 48 F CBI: 60 M 45 F	NA	0.5% schizophrenia 32.1% mood disorders 15.2% personality disorders 2.3% neurotic and somatoform disorders	• combined group behavioral intervention (105, 8 groups) •group interactional psychotherapy (110, 8 groups)	• weekly individual consultations with the referring therapist to record lapses and relapses (described as relapse: temporary alcohol intake that the subject was unable to control and which led to inpatient detoxification) and any treatment conclusions (drop outs)	weekly	SUBSTANCE USE: • PARTECIPATION: • SYMPTOMATIC CONDITIONS: X

Ojehagen et al [39]	Randomized Controlled Trial	1/2 years	72	outpatient	alcohol	37 (9)	60 M 12 F	NA	NA	• psychiatric treatment (supportive-expressive psychodynamic counseling - 1 year (12) • psychiatric treatment (expressive supportive psychodynamic counseling - 2 year (24) • multimodal behavior therapy - 1 year (24) • multimodal behavior therapy - 2 years (12)	• measure of alcohol consumption by liver function tests (number of days of abuse, i.e., more than 4 drinks in a row. Defined as favorable with <14 days of abuse during the year of follow up)	baseline, 12, 24, 36 months	SUBSTANCE USE: • PARTECIPATION: • SYMPTOMATIC CONDITIONS: X

Rounsaville et al [41]	Randomized Controlled Trial	24 weeks	72	outpatient	opioids	57% > 27 years	61% M 39% F	whites: 75% IPT 40% LCG	major depression(%): 18.9 IPG 11.4 LCG minor depression(%): 2.7 IPG 0 LCG intermittent depression(%): 8.1 IPG 14.3 LCG labile personality (%): 24.3 IPG 17.1 LCG cyclothymic personality disorder(%): 2.7 IPG 0 LCG generalized anxiety disorder(%): 2.7 IPG 2.9 LCG phobic disorder(%): 2.7 IPG 2.9 LCG mixed personality	• brief psychodynamic interpersonal therapy (37) • low contact treatment (35)	• evaluation of the program (dropouts (voluntary interruption or need for interventions not permitted by the study treatment protocol), urine tests, number of arrests, occupation and use of alcohol) • psychological symptomatology (Raskin depression scale, Symptom checklist 90) • personality assessment (Rotter's locus of control measure, Maudsley personality inventory) • social functioning (social adjustment scale self-report) • changes in target symptoms (Kiresuk TJ, Sherman RE: Goal attainment scaling, clinical assessment of symptom severity [63])	baseline, 12 and 24 weeks	SUBSTANCE USE: • PARTECIPATION: • SYMPTOMATIC CONDITIONS: •

									disorder(%): 8.1 IPG 5.7 LCG antisocial personality disorder(%): 10.8 IPG 22.8 LCG borderline personality disorder(%): 10.8 IPG 8.6 LCG somatoform disorders (briquet's syndrome)(%): 0 IPG 8.6 LCG schizotypal personality disorder(%): 5.5 IPG 5.7 LPG narcissistic personality disorder(%): 2.7 IPG 0 LCG				
Sandahl et al [38]	Randomized Controlled Trial	18 months	59	outpatient	alcohol	PD: 34-57 (mean 46) CB: 35-67	PD: 32% M 68% F CB: 42%	NA	NA	•time-limited psychodynamic group therapy (25, 5 groups)	• alcohol use (Timeline Follow Back Interview, Quantity Frequency Symptom Scale)	baseline and 18 months	SUBSTANCE USE: • PARTECIPATION: X

						(mean 46)	M 58% F			•time-limited cognitive behavioral group therapy (24, 5 groups)	• psychopathology and personality functioning (Symptom Check List, Tennessee Self-Concept Scale)		SYMPTOMATIC CONDITIONS •
Shaffer, LaSalvia & Stein [18]	Randomized Controlled Trial	5 months	59	outpatient	opioids	35.92 PG: 35.53 HY: 36.25	PG: 19M 10F HY: 16M 14F	whites: 50 blacks: 7 Hispanics: 1 Asians: 1	NA	• psychodynamic group therapy (30) • Hertha Yoga therapy (29)	• basic psychological measures (Revised Symptom Check List) • indices of addictive behavior (Addiction Severity Index)	baseline and 5 months	SUBSTANCE USE: • PARTECIPATION: • SYMPTOMATIC CONDITIONS: X
Woody et al [42]	Randomized Controlled Trial	6 months	110	outpatient	opioids	SE: 31 (25-37) CB: 29 (23-35) CG: 29 (25-34)	110 M	whites (%): 41 SE 33 CB 41 DC blacks (%): 59 SE 67 CB 59 DC	major depressive disorder: 47 minor depressive disorder: 8 intermittent depressive disorder: 13 labile personality: 10 cyclothymic disorder: 11 hypomanic	• drug counseling (32) • supportive-expressive therapy + drug counseling (32) • cognitive-behavioral therapy + drug counseling (39)	• psychological tests (Beck Depression Inventory, Maudsley Personality Inventory, Hopkins Symptom Checklist—90 items, Shipley Institute of Living Scale) • structured interviews (Schedule for Affective Disorders and Schizophrenia -Lifetime & -Change, Addiction	start treatment, 1 month before conclusion and after 24 weeks	SUBSTANCE USE: • PARTECIPATION: X SYMPTOMATIC CONDITIONS: X

									disorder: 21 manic disorder: 1 bipolar disorder 2:11 generalized anxiety disorder: 3 panic disorder:0 phobic disorder:4 OCD: 2 alcoholism: 26 antisocial personality disorder: 16 schizotypal features: 6 other psychiatric disorders: 1		Severity Index) • evaluation of the program (methadone dosages, ancillary drugs, urinalysis)		
Woody, McLellan, Luborsky & O'Brien. [53]	Randomized Controlled Trial	6 months	93	outpatient	opioids	NA	93 M	same as Woody (1983)	same as Woody (1983)	• drug counseling (32) • supportive-expressive therapy + drug counseling (32)	• psychological tests (Beck Depression Inventory, Maudsley Personality Inventory, Hopkins Symptom Checklist—90 items, Shipley	baseline, 7 months, and 12 months	SUBSTANCE USE: • PARTECIPATION: X SYMPTOMATIC CONDITIONS: •

										• cognitive-behavioral therapy + drug counseling (39)	Institute of Living Scale) • structured interviews (Schedule for Affective Disorders and Schizophrenia -Lifetime & -Change, Addiction Severity Index)		
Woody, McLellan, Luborsky & O'Brien [20]	Randomized Controlled Trial	6 months	110	outpatient	opioids	41 (7)	76 M 34 F	Caucasians: 62 African Americans: 48	depressive symptoms: 72 attempted suicide: 27	• counseling + drug counseling (62) • counseling + supportive-expressive therapy (31)	• evaluation of the program (dosages of methadone, auxiliary psychotropic drugs, urinalysis) • psychological tests (Beck Depression Inventory, Maudsley Personality Inventory, Hopkins Symptom Checklist—90 items, Shipley Institute of Living Scale) • structured interviews (Schedule for Affective Disorders and Schizophrenia -Lifetime & -Change, Addiction Severity Index)	baseline 1 month and 6 months	SUBSTANCE USE: • PARTECIPATION: • SYMPTOMATIC CONDITIONS: •

• evaluation of the program (dosages of methadone, auxiliary psychotropic drugs, urinalysis)

Table S2: Statistical analyses carried out on the sample using alcohol, respectively in relation to “substance use”.

STUDY NAME	COMPARISON	OUTCOME	TIME POINT	DATA FORMAT	DT ALCOHOL USE RATE	DT TOTAL N	C ALCOHOL USE RATE	C TOTAL N	DT MEAN	DT STD-DEV	DT SAMPLE SIZE	C MEAN	C STD-DEV	C SAMPLE SIZE	EFFECT DIRECTION	ODDS RATIO	LOG ODDS RATIO	STD ERR
Ojahagen et al [39]	1Y PT vs 1Y MBT	14 Max misuse days /Y	Blank	Cohort 2x2 (rates)	0.44	9.00	0.68	22.00	-	-	-	-	-	-	-	0.37	-0.99	0.81
Ojahagen et al [39]	2Y PT vs 2Y MBT	14 Max misuse days /Y	Blank	Cohort 2x2 (rates)	0.40	20.00	0.41	12.00	-	-	-	-	-	-	-	0.96	-0.04	0.74
Ojahagen et al [39]	DDP vs TAU	% with alcohol misuse	0 months	Cohort 2x2 (rates)	0.67	15.00	0.67	15.00	-	-	-	-	-	-	-	1.00	0.00	0.78
Gregory et al [35]	DDP vs TAU	% with alcohol misuse	12 months	Cohort 2x2 (rates)	0.30	15.00	0.44	15.00	-	-	-	-	-	-	-	0.55	-0.61	0.77
Gregory et al [35]	DDP vs TAU	% with alcohol misuse	6 months	Cohort 2x2 (rates)	0.45	15.00	0.45	15.00	-	-	-	-	-	-	-	1.00	0.00	0.73
Gregory et al [28]	DDP vs TAU	% heavy drinking days	12 months	Independent groups (means, SD's)	-	-	-	-	1.70	3.20	11.00	17.40	33.00	13.00	1.00	0.31	-1.16	0.76

Gregory et al [28]	DDP vs TAU	% heavy drinking days	30 months	Independent groups (means, SD's)	-	-	-	-	0.80	1.50	11.00	9.20	16.00	13.00	1.00	0.28	-1.28	0.77
Nyhuis et al [37]	PGA vs BGT	relapse rate	Blank	Cohort 2x2 (rates)	0.34	110.00	0.50	105.00	-	-	-	-	-	-	-	0.52	-0.66	0.28
Ito, Donovan & Hall [36]	IP vs RP	drinking days at follow up	Blank	Independent groups (means, SD's)	-	-	-	-	7.44	17.28	16.00	3.67	7.91	15.00	1.00	1.65	0.50	0.66
Ito, Donovan & Hall [36]	IP vs RP	one month abstinence	Blank	Cohort 2x2 (rates)	0.27	15.00	0.24	17.00	-	-	-	-	-	-	-	1.19	0.17	0.82
Ito, Donovan & Hall [36]	IP vs RP	six-month abstinence	Blank	Cohort 2x2 (rates)	0.58	19.00	0.50	18.00	-	-	-	-	-	-	-	1.38	0.32	0.66
Sandahl et al [38]	PD vs CBT	number of abstinent days	Blank	Independent groups (means, SD's)	-	-	-	-	74.00	24.00	21.00	54.00	37.00	21.00	1.00	3.20	1.16	0.57
Sandahl et al [38]	PD vs CBT	sobriety (reversed)	Blank	Cohort 2x2 (rates)	0.86	21.00	0.56	23.00	-	-	-	-	-	-	-	4.83	1.57	0.76

Independent groups mean and SD and cohort 2x2 rates have been gathered as data for effect size calculation. Time points and outcome measures have been reported. DT= dynamic treatment; C= control treatment

Table S3: Statistical analyses carried out on the sample using alcohol, respectively in relation to “participation”.

STUDY NAME	COMPARISON	TIME POINT	DATA FORMAT	DT DROPOUT	DT TOTAL N	C DROPOUT	C TOTAL N	ODDS RATIO	LOG ODDS RATIO	STD ERR

Ojahagen et al [39]	1Y PT vs 1Y MBT	Blank	Cohort 2x2 (events)	3	12	7	24	0.81	-0.21	0.803
Ojahagen et al [39]	2Y PT vs 2Y MBT	Blank	Cohort 2x2 (events)	9	24	6	12	0.60	-0.51	0.71
Gregory et al [35]	DDP vs TAU	Blank	Cohort 2x2 (events)	5	15	6	15	0.75	-0.29	0.76
Nyhuis et al [37]	PGT vs BGT	Blank	Cohort 2x2 (events)	5	110	7	105	0.67	-0.41	0.60
Ito, Donovan & Hall [36]	IP vs RP	Blank	Cohort 2x2 (events)	4	19	8	20	0.40	-0.92	0.72

C cohort 2x2 events have been gathered as data for effect size calculation. Time points and outcome measures have been reported. DT= dynamic treatment; C= control treatment.

Table S4: Statistical analyses carried out on the sample using alcohol, respectively in relation to "other symptomatic conditions".

STUDY NAME	COMPARISON	OUTCOME	TIME POINT	DATA FORMAT	DT MEAN	DT STD-DEV	DT SAMPLE SIZE	C MEAN	C STD-DEV	C SAMPLE SIZE	EFFECT DIRECTION	STD DIFF IN MEANS	STD ERR	HEDGES'S G	STD ERR 2	DIFFERENCE IN MEANS	STD ERR 3
Hoyer et al [44]	PD vs CBT	global severity index	1	Independent groups (means, SD's)	0.53	0.40	40	0.81	0.68	47	1	-0.49	0.22	-0.49	0.22	-0.28	0.12
Hoyer et al [44]	PD vs CBT	global severity index	2	Independent groups (means, SD's)	0.38	0.36	40	0.55	0.57	47	1	-0.35	0.22	-0.35	0.21	-0.17	0.10
Hoyer et al [44]	PD vs CBT	global severity index	3	Independent groups (means, SD's)	0.37	0.39	40	0.51	0.57	47	1	-0.28	0.22	-0.28	0.21	-0.14	0.10
Hoyer et al [44]	PD vs CBT	global severity index	4	Independent groups	0.37	0.45	40	0.44	0.53	47	1	-0.14	0.22	-0.14	0.21	-0.07	0.11

				(means, SD's)												
Gregory, DeLucia-Deranja & Mogle [28]	DDP vs OCC	beck depression inventory	6 months prior to enrollment	Independent groups (means, SD's)	31.10	13.80	15	25.60	8.20	15	1	0.48	0.37	0.47	0.36	5.50	4.14
Gregory, DeLucia-Deranja & Mogle [28]	DDP vs OCC	beck depression inventory	6 months	Independent groups (means, SD's)	23.80	11.40	15	21.60	11.40	15	1	0.19	0.37	0.19	0.36	2.20	4.16
Gregory, DeLucia-Deranja & Mogle [28]	DDP vs OCC	beck depression inventory	6 to12 months	Independent groups (means, SD's)	21.00	11.40	15	25.10	6.40	15	1	-0.44	0.37	-0.43	0.36	-4.10	3.38
Gregory, DeLucia-Deranja & Mogle [28]	DDP vs OCC	beck depression inventory	18-30 months follow up	Independent groups (means, SD's)	16.00	11.50	15	23.80	10.60	15	1	-0.71	0.38	-0.69	0.37	-7.80	4.04
Gregory, DeLucia-Deranja & Mogle [28]	DDP vs OCC	borderline evaluation severity index	6 months prior to enrollment	Independent groups (means, SD's)	48.30	10.00	15	44.00	7.50	15	1	0.49	0.37	0.47	0.36	4.30	3.23

Gregory, DeLucia-Deranja & Mogle [28]	DDP vs OCC	borderline evaluation severity index	6 months	Independent groups (means, SD's)	39.30	10.00	15	40.50	12.20	15	1	-0.11	0.37	-0.10	0.36	-1.20	4.07
Gregory, DeLucia-Deranja & Mogle [28]	DDP vs OCC	borderline evaluation severity index	6 to12 months	Independent groups (means, SD's)	33.60	12.40	15	38.40	8.60	15	1	-0.45	0.37	-0.44	0.36	-4.80	3.90
Gregory, DeLucia-Deranja & Mogle [28]	DDP vs OCC	borderline evaluation severity index	18-30 months follow up	Independent groups (means, SD's)	31.50	12.10	15	38.10	9.50	15	1	-0.61	0.37	-0.59	0.36	-6.60	3.97
Gregory, DeLucia-Deranja & Mogle [28]	DDP vs OCC	dissociative experiences scale	6 months prior to enrollment	Independent groups (means, SD's)	35.00	22.10	15	27.40	12.10	15	1	0.43	0.37	0.42	0.36	7.60	6.51
Gregory, DeLucia-Deranja & Mogle [28]	DDP vs OCC	dissociative experiences scale	6 months	Independent groups (means, SD's)	31.40	18.40	15	24.80	18.30	15	1	0.36	0.37	0.35	0.36	6.60	6.70
Gregory, DeLucia-	DDP vs OCC	dissociative experiences scale	6 to12 months	Independent groups	27.70	21.40	15	22.30	20.60	15	1	0.26	0.37	0.25	0.36	5.40	7.67

Deranja & Mogle [28]				(means, SD's)												
Gregory, DeLucia-Deranja & Mogle [28]	DDP vs OCC	dissociative experiences scale	18-30 months follow up	Independent groups (means, SD's)	28.40	28.90	15	28.60	18.70	15	1	-0.01	0.37	-0.01	0.36	-0.20	8.89
Sandahl, et al [38]	DGT vs CBT	global severity index	before	Independent groups (means, SD's)	7.80	13.00	21	8.80	19.00	21	1	-0.06	0.31	-0.06	0.30	-1.00	5.02
Sandahl, et al [38]	DGT vs CBT	global severity index	after 15 month follow up	Independent groups (means, SD's)	1.10	0.40	21	1.20	0.60	21	1	-0.20	0.31	-0.19	0.30	-0.10	0.16
I Ito, Donovan & Hall [36]	IP vs RP	impairment due to alcohol	pre	Independent groups (means, SD's)	1.63	2.19	16	1.87	2.50	15	1	-0.10	0.36	-0.10	0.35	-0.24	0.84
Ito, Donovan & Hall [36]	IP vs RP	impairment due to alcohol	6 months follow up	Independent groups (means, SD's)	4.06	1.91	16	3.53	2.07	15	1	0.27	0.36	0.26	0.35	0.53	0.71

Independent groups means and SD have been gathered as data for effect size calculation. Time points and outcome measures have been reported. DT= dynamic treatment; C= control treatment**.**

Table S5: Statistical analyses carried out on the sample using cocaine, respectively in relation to “substance use”.

STUDY NAME	COMPARISON	OUTCOME	TIME POINT	DATA FORMAT	DT COCAINE USE RATE	DT TOTAL N	C COCAINE USE RATE	C TOTAL N	ODDS RATIO	LOG ODDS RATIO	STD ERR

Crits-Christoph et al [15]	SE vs CT	Abstinence R	1 month	Cohort 2x2 (rates)	0.40	124	0.46	119	0.78	-0.25	0.26
Crits-Christoph et al [15]	SE vs CT	Abstinence R	2 months	Cohort 2x2 (rates)	0.68	124	0.64	119	1.19	0.17	0.27
Crits-Christoph et al [15]	SE vs CT	Abstinence R	3 months	Cohort 2x2 (rates)	0.82	124	0.77	119	1.37	0.32	0.32
Crits-Christoph et al [15]	SE VS GDC	Abstinence R	1 month	Cohort 2x2 (rates)	0.40	124	0.42	123	0.92	-0.09	0.26
Crits-Christoph et al [15]	SE VS GDC	Abstinence R	2 months	Cohort 2x2 (rates)	0.68	124	0.58	123	1.53	0.43	0.27
Crits-Christoph et al [15]	SE VS GDC	Abstinence R	3 months	Cohort 2x2 (rates)	0.82	124	0.73	123	1.72	0.54	0.31
Crits-Christoph et al [15]	SE vs IDC	Abstinence R	1 month	Cohort 2x2 (rates)	0.40	124	0.29	121	1.66	0.51	0.27
Crits-Christoph et al [15]	SE vs IDC	Abstinence R	2 months	Cohort 2x2 (rates)	0.68	124	0.52	121	1.97	0.68	0.26
Crits-Christoph et al [15]	SE vs IDC	Abstinence R	3 months	Cohort 2x2 (rates)	0.82	124	0.62	121	2.85	1.05	0.30
Carrol, Rounsaville & Gawin [40]	IPT vs RPT	Abstinence R	Blank	Cohort 2x2 (rates)	0.67	21	0.43	21	2.67	0.98	0.64
Carrol, Rounsaville & Gawin [40]	IPT vs RPT	Recovery R	Blank	Cohort 2x2 (rates)	0.89	21	0.57	21	6.08	1.80	0.83

Cohort 2x2 rates have been gathered as data for effect size calculation. Time points and outcome measures have been reported. DT= dynamic treatment; C= control treatment

Table S6: Statistical analyses carried out on the sample using cocaine, respectively in relation to "participation".

STUDY NAME	COMPARISON	OUTCOME	TIME POINT	DATA FORMAT	DT DROPOUT	DT TOTAL N	C DROPOUT	C TOTAL N	ODDS RATIO	LOG ODDS RATIO	STD ERR
Crits-Christoph et al [15]	SE vs CT	participation	Blank	Cohort 2x2 (events)	83	124	79	119	1.03	0.02	0.27

Crits-Christoph et al [15]	SE vs GDC	participation	Blank	Cohort 2x2 (events)	83	124	95	123	0.60	-0.52	0.29
Crits-Christoph et al [15]	SE vs IDC	participation	Blank	Cohort 2x2 (events)	83	124	93	121	0.61	-0.50	0.29
Carrol, Rounsaville & Gawin [40]	IPT vs RPT	participation	Blank	Cohort 2x2 (events)	13	21	7	21	3.25	1.18	0.65

Cohort 2x2 events have been gathered as data for effect size calculation. Time points and outcome measures have been reported. DT= dynamic treatment; C= control treatment

Table S7: Statistical analyses carried out on the sample using opiates, respectively in relation to “substance use”.

STUDY NAME	COMPARISON	OUTCOME	TIME POINT	DATA FORMAT	DT MEAN	DT STD-DEV	DT SAMPLE SIZE	C MEAN	C STD-DEV	C SAMPLE SIZE	EFFECT DIRECTION	DT OPIATE USE	DT TOTAL N	C OPIATE USE	C TOTAL N	STD DIFF IN MEANS	STD ERR	HEDGES'S G	STD ERR3	DIFFERENCE IN MEANS	STD ERR2
Woody et al [53]	SE vs CB	ASI drug	0 months	Independent groups (means, SD's)	177.00	21.00	25	204.00	18.00	31	1	-	-	-	-	-1.39	0.30	-1.37	0.30	-27.00	5.21
Woody et al [53]	SE vs CB	ASI drug	12 months	Independent groups (means, SD's)	158.00	17.00	25	96.00	11.00	31	1	-	-	-	-	4.43	0.50	4.37	0.49	62.00	3.76
Woody et al [53]	SE vs DC	ASI drug	0 months	Independent groups (means, SD's)	177.00	21.00	25	205.00	12.00	31	1	-	-	-	-	-1.69	0.31	-1.66	0.31	-28.00	4.47
Woody et al [53]	SE vs DC	ASI drug	12 months	Independent groups (means, SD's)	158.00	17.00	25	116.00	12.00	31	1	-	-	-	-	2.91	0.38	2.87	0.38	42.00	3.88
Woody et al [52]	SE vs CB	ASI drug	0 months	Independent groups	0.25	0.13	31	0.26	0.11	34	1	-	-	-	-	-0.11	0.25	-0.11	0.25	-0.01	0.03

				(means, SD's)																	
Woody et al [52]	SE vs CB	ASI drug	7 months	Independent groups (means, SD's)	0.17	0.13	31	0.18	0.11	34	1	-	-	-	-	-0.08	0.25	-0.08	0.25	-0.01	0.03
Woody et al [52]	SE vs DC	ASI drug	0 months	Independent groups (means, SD's)	0.25	0.13	31	0.24	0.13	35	1	-	-	-	-	0.05	0.25	0.05	0.24	0.01	0.03
Woody et al [52]	SE vs DC	ASI drug	7 months	Independent groups (means, SD's)	0.17	0.13	31	0.18	0.13	35	1	-	-	-	-	-0.02	0.25	-0.02	0.24	0.00	0.03
Woody et al [20]	SE vs DC	ASI drug	1 months	Independent groups (means, SD's)	0.36	0.13	82	0.37	0.12	41	1	-	-	-	-	-0.05	0.19	-0.05	0.19	-0.01	0.02
Woody et al [20]	SE vs DC	ASI drug	2 months	Independent groups (means, SD's)	0.30	0.12	82	0.31	0.11	41	1	-	-	-	-	-0.10	0.19	-0.10	0.19	-0.01	0.02
Woody et al [20]	SE vs DC	ASI drug	3 months	Independent groups (means, SD's)	0.22	0.15	82	0.29	0.19	41	1	-	-	-	-	-0.45	0.19	-0.45	0.19	-0.07	0.03
Rounsaville et al [41]	IPG vs LCG	urine positive	0 months	Cohort 2x2 (Events)	-	-	-	-	-	-	-	0.47	22	1.05	28	-0.32	0.98	-0.31	0.97	-	-
Rounsaville et al [41]	IPG vs LCG	urine positive	12 months	Cohort 2x2 (Events)	-	-	-	-	-	-	-	0.86	20	0.90	24	0.08	0.85	0.08	0.83	-	-
Rounsaville et al [41]	IPG vs LCG	urine positive	24 months	Cohort 2x2 (Events)	-	-	-	-	-	-	-	1.43	21	1.25	23	0.13	0.70	0.13	0.68	-	-

Independent group mean and SD and Cohort 2x2 events have been gathered as data for effect size calculation. Time points and outcome measures have been reported. DT= dynamic treatment; C= control treatment.

Table S8: Statistical analyses carried out on the sample using opiates, respectively in relation to "participation".

STUDY NAME	COMPARISON	OUTCOME	TIME POINT	DATA FORMAT	DT DROPOUT	DT TOTAL N	C DROPOUT	C TOTAL N	DT MEAN	DT STD-DEV	DT SAMPLE SIZE	C MEAN	C STD-DEV	C SAMPLE SIZE	EFFECT DIRECTION	ODDS RATIO	LOG ODDS RATIO	STD ERR
Rounsaville et al [41]	IPG vs LCG	dropout	17-24 weeks	Cohort 2x2 (Events)	2	16	2	21	-	-	-	-	-	-	-	1.36	0.31	1.06
Rounsaville et al [41]	IPG vs LCG	dropout	1-8 weeks	Cohort 2x2 (Events)	15	37	7	35	-	-	-	-	-	-	-	2.73	1.00	0.54
Rounsaville et al [41]	IPG vs LCG	dropout	9-16 weeks	Cohort 2x2 (Events)	6	22	7	28	-	-	-	-	-	-	-	1.13	0.12	0.65
Shaffer, LaSalvia & Stein [18]	PDT vs AMT	retention	Blank	Cohort 2x2 (Events)	10	30	8	29	-	-	-	-	-	-	-	1.31	0.27	0.57
Woody et al [20]	SE vs DC	attendance	Blank	Independent groups (means, SD's)	-	-	-	-	26	6	82	23	6	41	1	2.48	0.91	0.35

Independent group mean and SD and Cohort 2x2 events have been gathered as data for effect size calculation. Time points and outcome measures have been reported. DT= dynamic treatment; C= control treatment

Table S9: Statistical analyses carried out on the sample using opiates, respectively in relation to "other symptomatic conditions".

STUDY NAME	COMPARISON	OUTCOME	TIME POINT	DATA FORMAT	DT MEAN	DT STD-DEV	DT SAMPLE SIZE	C MEAN	C STD-DEV	C SAMPLE SIZE	EFFECT DIRECTION	STD DIFF IN MEANS	STD ERR	HEDGES'S G	STD ERR 2	DIFFERENCE IN MEANS	STD ERR 3

Woody et al [53]	SE vs CB	ASI psych	0 months	Independent groups (means, SD's)	171.00	38.00	28	156.00	44.00	34	1	0.36	0.26	0.36	0.25	15.00	10.57
Woody et al [53]	SE vs CB	ASI psych	7 months	Independent groups (means, SD's)	80.00	19.00	28	75.00	20.00	34	1	0.26	0.26	0.25	0.25	5.00	4.99
Woody et al [53]	SE vs CB	BDI	0 months	Independent groups (means, SD's)	15.00	3.00	28	14.00	2.00	34	1	0.40	0.26	0.39	0.25	1.00	0.64
Woody et al [53]	SE vs CB	BDI	7 months	Independent groups (means, SD's)	9.00	2.00	28	9.00	2.00	34	1	0.00	0.26	0.00	0.25	0.00	0.51
Woody et al [53]	SE vs CB	GAS	0 months	Independent groups (means, SD's)	66.00	7.00	28	57.00	8.00	34	1	1.19	0.28	1.17	0.27	9.00	1.93
Woody et al [53]	SE vs CB	GAS	7 months	Independent groups (means, SD's)	78.00	7.00	28	69.00	6.00	34	1	1.39	0.28	1.37	0.28	9.00	1.65
Woody et al [53]	SE vs CB	MPI neuroticism	0 months	Independent groups (means, SD's)	26.00	4.00	28	26.00	4.00	34	1	0.00	0.26	0.00	0.25	0.00	1.02
Woody et al [53]	SE vs CB	MPI neuroticism	7 months	Independent groups (means, SD's)	19.00	2.00	28	21.00	4.00	34	1	-0.61	0.26	-0.61	0.26	-2.00	0.83
Woody et al [53]	SE vs CB	SADS anxiety	0 months	Independent groups	18.00	3.00	28	20.00	4.00	34	1	-0.56	0.26	-0.55	0.26	-2.00	0.91

				(means, SD's)													
Woody et al [53]	SE vs CB	SADS anxiety	7 months	Independent groups (means, SD's)	18.00	4.00	28	17.00	3.00	34	1	0.29	0.26	0.28	0.25	1.00	0.89
Woody et al [53]	SE vs CB	SADS depression	0 months	Independent groups (means, SD's)	20.00	5.00	28	23.00	5.00	34	1	-0.60	0.26	-0.59	0.26	-3.00	1.28
Woody et al [53]	SE vs CB	SADS depression	7 months	Independent groups (means, SD's)	16.00	4.00	28	20.00	5.00	34	1	-0.87	0.27	-0.86	0.26	-4.00	1.17
Woody et al [53]	SE vs CB	SCL 90 total	0 months	Independent groups (means, SD's)	66.00	10.00	28	56.00	7.00	34	1	1.18	0.28	1.16	0.27	10.00	2.16
Woody et al [53]	SE vs CB	SCL 90 total	7 months	Independent groups (means, SD's)	29.00	5.00	28	47.00	4.00	34	1	-4.02	0.44	-3.97	0.44	-18.00	1.14
Woody et al [53]	SE vs DC	ASI psych	0 months	Independent groups (means, SD's)	171.00	38.00	28	160.00	26.00	31	1	0.34	0.26	0.34	0.26	11.00	8.41
Woody et al [53]	SE vs DC	ASI psych	7 months	Independent groups (means, SD's)	80.00	19.00	28	111.00	31.00	31	1	-1.19	0.28	-1.18	0.28	-31.00	6.78
Woody et al [53]	SE vs DC	BDI	0 months	Independent groups (means, SD's)	15.00	3.00	28	14.00	3.00	31	1	0.33	0.26	0.33	0.26	1.00	0.78

Woody et al [53]	SE vs DC	BDI	7 months	Independent groups (means, SD's)	9.00	2.00	28	11.00	6.00	31	1	-0.44	0.26	-0.43	0.26	-2.00	1.19
Woody et al [53]	SE vs DC	GAS	0 months	Independent groups (means, SD's)	66.00	7.00	28	66.00	9.00	31	1	0.00	0.26	0.00	0.26	0.00	2.12
Woody et al [53]	SE vs DC	GAS	7 months	Independent groups (means, SD's)	78.00	7.00	28	68.00	8.00	31	1	1.33	0.29	1.31	0.28	10.00	1.97
Woody et al [53]	SE vs DC	MPI neuroticism	0 months	Independent groups (means, SD's)	26.00	4.00	28	28.00	4.00	31	1	-0.50	0.26	-0.49	0.26	-2.00	1.04
Woody et al [53]	SE vs DC	MPI neuroticism	7 months	Independent groups (means, SD's)	19.00	2.00	28	26.00	4.00	31	1	-2.18	0.33	-2.15	0.32	-7.00	0.84
Woody et al [53]	SE vs DC	SADS anxiety	0 months	Independent groups (means, SD's)	18.00	3.00	28	18.00	3.00	31	1	0.00	0.26	0.00	0.26	0.00	0.78
Woody et al [53]	SE vs DC	SADS anxiety	7 months	Independent groups (means, SD's)	18.00	4.00	28	22.00	4.00	31	1	-1.00	0.28	-0.99	0.27	-4.00	1.04
Woody et al [53]	SE vs DC	SADS depression	0 months	Independent groups (means, SD's)	20.00	5.00	28	21.00	4.00	31	1	-0.22	0.26	-0.22	0.26	-1.00	1.17
Wood, et al [53]	SE vs DC	SADS depression	7 months	Independent groups	16.00	4.00	28	20.00	2.00	31	1	-1.29	0.29	-1.27	0.28	-4.00	0.81

				(means, SD's)													
Woody et al [53]	SE vs DC	SCL 90 total	0 months	Independent groups (means, SD's)	66.00	10.00	28	72.00	12.00	31	1	-0.54	0.27	-0.53	0.26	-6.00	2.89
Woody et al [53]	SE vs DC	SCL 90 total	7 months	Independent groups (means, SD's)	29.00	5.00	28	54.00	11.00	31	1	-2.88	0.37	-2.84	0.37	-25.00	2.27
Woody et al [20]	SE vs DC	ASI psych	0 months	Independent groups (means, SD's)	0.42	0.14	82	0.43	0.21	41	1	-0.07	0.19	-0.07	0.19	-0.01	0.03
Woody et al [20]	SE vs DC	ASI psych	1 month s	Independent groups (means, SD's)	0.34	0.13	82	0.32	0.26	41	1	0.10	0.19	0.10	0.19	0.02	0.03
Woody et al [20]	SE vs DC	ASI psych	6 months	Independent groups (means, SD's)	0.29	0.24	82	0.34	0.20	41	1	-0.18	0.19	-0.18	0.19	-0.04	0.04
Woody et al [20]	SE vs DC	BDI	0 months	Independent groups (means, SD's)	21.00	9.00	82	24.00	10.00	41	1	-0.32	0.19	-0.32	0.19	-3.00	1.79
Woody et al [20]	SE vs DC	BDI	1 months	Independent groups (means, SD's)	16.00	11.00	82	17.00	10.00	41	1	-0.09	0.19	-0.09	0.19	-1.00	2.04
Woody et al [20]	SE vs DC	BDI	6 months	Independent groups (means, SD's)	15.00	10.00	82	20.00	15.00	41	1	-0.42	0.19	-0.42	0.19	-5.00	2.27

Woody et al [20]	SE vs DC	MPI neuroticism	0 months	Independent groups (means, SD's)	32.00	6.00	82	32.00	7.00	41	1	0.00	0.19	0.00	0.19	0.00	1.21
Woody et al [20]	SE vs DC	MPI neuroticism	1 months	Independent groups (means, SD's)	28.00	10.00	82	30.00	13.00	41	1	-0.18	0.19	-0.18	0.19	-2.00	2.12
Woody et al [20]	SE vs DC	MPI neuroticism	6 months	Independent groups (means, SD's)	28.00	11.00	82	37.00	14.00	41	1	-0.75	0.20	-0.74	0.20	-9.00	2.31
Woody et al [20]	SE vs DC	SCL 90 total	0 months	Independent groups (means, SD's)	106.00	61.00	82	120.00	73.00	41	1	-0.21	0.19	-0.21	0.19	-14.00	12.4
Woody et al [20]	SE vs DC	SCL 90 total	1 months	Independent groups (means, SD's)	78.00	53.00	82	77.00	59.00	41	1	0.02	0.19	0.02	0.19	1.00	10.5
Woody et al [20]	SE vs DC	SCL 90 total	6 months	Independent groups (means, SD's)	95.00	51.00	82	109.00	68.00	41	1	-0.24	0.19	-0.24	0.19	-14.00	10.9

Independent group mean and SD have been gathered as data for effect size calculation. Time points and outcome measures have been reported. DT= dynamic treatment; C= control treatment
